# Bringing Cardiovascular Comorbidities in Acromegaly to an Update. How Should We Diagnose and Manage Them?

**DOI:** 10.3389/fendo.2019.00120

**Published:** 2019-03-07

**Authors:** Ana M. Ramos-Leví, Mónica Marazuela

**Affiliations:** Department of Endocrinology, Instituto de Investigación Princesa, Hospital Universitario La Princesa, Universidad Autónoma, Madrid, Spain

**Keywords:** acromegaly, acromegaly cardiovascular comorbidities, acromegalic cardiomyopathy, hypertension, valve disease, somatostatin analogs, pegvisomant, radiotherapy

## Abstract

Patients with acromegaly frequently develop cardiovascular comorbidities, which significantly affect their morbidity and contribute to an increased all-cause mortality. In this regard, the most frequent complications that these patients may encounter include hypertension, cardiomyopathy, heart valve disease, arrhythmias, atherosclerosis, and coronary artery disease. The specific underlying mechanisms involved in the pathophysiology of these comorbidities are not always fully understood, but uncontrolled GH/IGF-I excess, age, prolonged disease duration, and coexistence of other cardio-vascular risk factors have been identified as significant influencing predisposing factors. It is important that clinicians bear in mind the potential development of cardiovascular comorbidities in acromegalic patients, in order to promptly tackle them, and avoid the progression of cardiac abnormalities. In many cases, this approach may be performed using straightforward screening tools, which will guide us for further diagnosis and management of cardiovascular complications. This article focuses on those cardiovascular comorbidities that are most frequently encountered in acromegalic patients, describes their pathophysiology, and suggests some recommendations for an early and optimal diagnosis, management and treatment.

## Introduction

Acromegaly is a rare chronic disease caused by GH hypersecretion, characterized by overgrowth of tissues, which leads to a considerable burden of complications and co-existing illnesses, such as cardiovascular, respiratory, endocrine, and metabolic morbidities (1–3).

Cardiovascular comorbidities are one of the most prevalent in patients with acromegaly. For instance, the prevalence of hypertension may reach up to 50% of patients with active disease. Patients also frequently develop atherosclerosis and coronary artery disease, septal hypertrophy, and left ventricular dysfunction. In fact, up to 20% of patients may develop symptomatic cardiac disease. The sinoatrial and atrioventricular nodes may also be affected, entailing cardiac arrhythmias and sudden death. In addition, together with these classical factor-derived cardiac alterations, a specific acromegalic cardiomyopathy has also been described (4). Duration of GH hypersecretion, age, and BMI have been identified as determinant factors for developing hypertension and cardiac abnormalities (5, 6).

The presence of cardiovascular comorbidities significantly increases the risk of morbidity and all-cause mortality, especially due to the frequently encountered concomitant problems, such as impaired glucose tolerance or diabetes, dyslipidemia and sleep apnea. In fact, the presence of any sort of cardiovascular disease at the time of diagnosis of acromegaly may triple the odds of hospitalization, and may account for as much as 60–100% of deaths in acromegalic patients within 15 years. Not surprisingly, cardiovascular comorbidities significantly increase the annual mean cost associated to acromegaly, despite specific cardiac improvement after effective GH and IGF-I control (7–12). Recently proposed instruments such as SAGIT (Signs and symptoms, Associated comorbidities, GH levels, IGF1 levels and Tumor profile) (13) and ACRODAT (Acromegaly Disease Activity Tool) (14), in addition to routine diagnostic methods, could possibly be used to specifically evaluate the presence of these comorbidities at the time of diagnosis of acromegaly, and also during follow-up (15).

In this article, we review the most relevant comorbidities related to the cardiovascular system that patients with acromegaly may encounter. Specifically, we will review the pathophysiology and underlying mechanisms involved in the development of hypertension, cardiomyopathy, valve disease, arrhythmias, atherosclerosis, and coronary heart disease, as well as the potential diagnostic tools that could be used in clinical practice for an early identification and diagnosis, emphasizing the strengths, and pitfalls of each of these diagnostic techniques. We also aim to provide some practical recommendations for their management and treatment from a clinical point of view. Finally, we will discuss how different acromegaly-directed treatments can affect these complications.

## An Overview of the Prevalence And Underlying Pathophysiology of the Main Cardiovascular Comorbidities in Patients With Acromegaly

**Figure 1:**
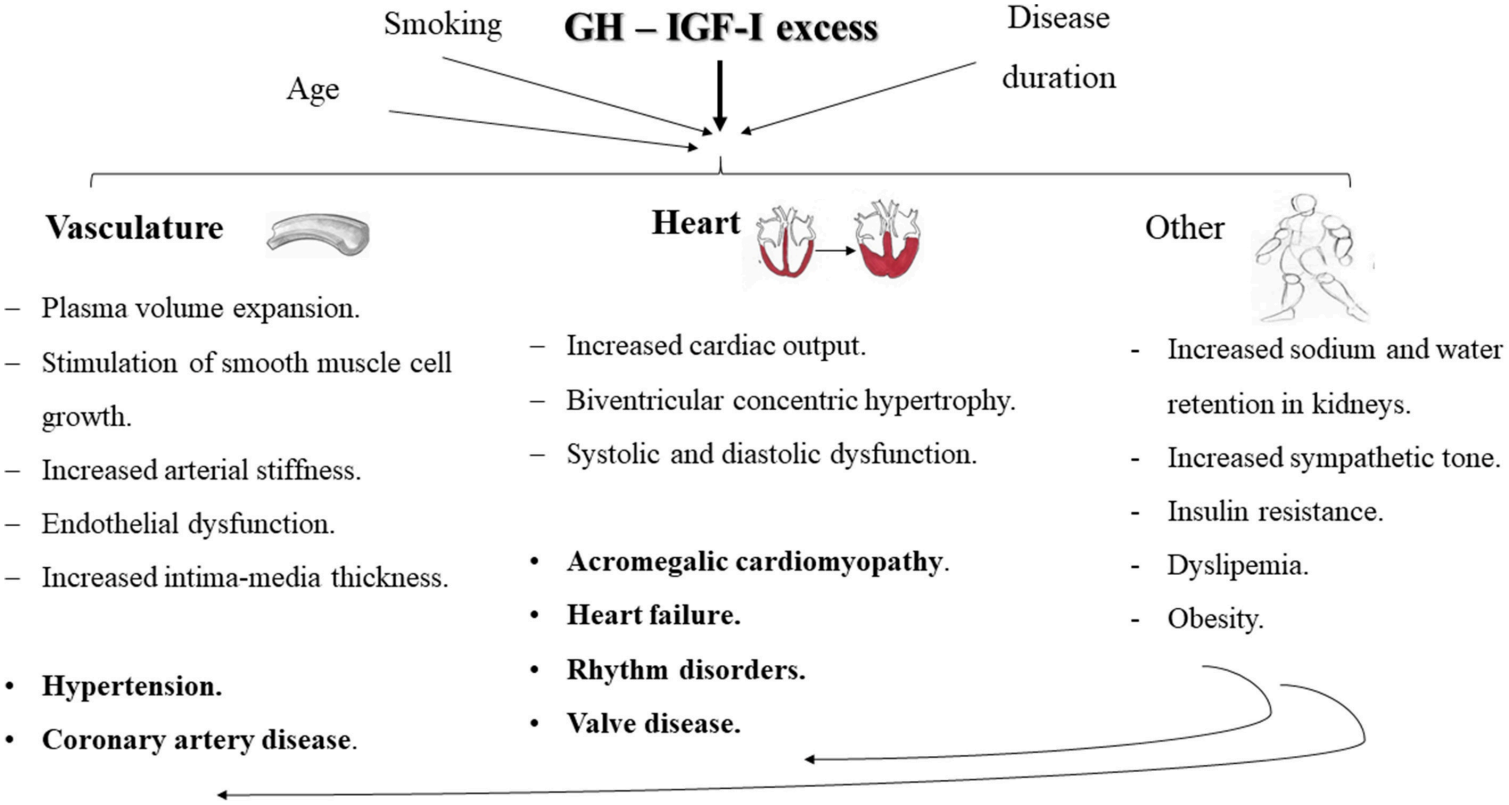
Schematic representation of the main cardiovascular comorbidities encountered in patients with acromegaly and the key mechanisms involved in their development. Chronic GH hypersecretion and/or IGF-I excess, exacerbated by the influence of other concomitant issues, such as disease duration, age and smoking, may lead to plasma volume expansion, stimulation of smooth muscle cell growth, increased arterial stiffness and endothelial dysfunction and increased intima-media thickness, which may all contribute to the development of hypertension and coronary artery disease. Besides, an increased vascular responsiveness to angiotensin II, sodium and water retention, and an increase in cardiac output, may also contribute to an increased peripheral resistance and exacerbate the development of hypertension. Increased cardiac output, biventricular concentric hypertrophy and systolic and diastolic dysfunction configure the key milestones in the development of acromegalic cardiomyopathy, eventually leading to heart failure. Other typical cardiac alterations in acromegalic patients include rhythm disorders and valve disease. Furthermore, there have been studies suggesting a role of the sympathetic tone in the development of comorbidities in acromegaly. Insulin resistance, obesity and an atherogenic lipid profile may aggravate the progression of cardiovascular alterations.

The main factors involved in the onset and progression of cardiovascular comorbidities in acromegaly are GH/IGF-I excess, disease duration, age, and other modifiable cardiovascular risk factors (including smoking, obesity, and dyslipidemia) (Figure 1). We will focus on the most relevant ones, given their impact on the individual's morbidity and mortality.

### Hypertension

Hypertension may be over-diagnosed if office measurements are used to estimate its prevalence, rather than ambulatory blood pressure monitoring (ABPM) (16). Nonetheless, it is one of the most frequent cardiovascular comorbidities observed in patients with acromegaly. In fact, although the global prevalence of high blood pressure in adults has been reported to be around 40% (17), acromegalic patients encounter a higher incidence, and a prevalence ranging from 18 to 60% (18). Disease duration may influence its development (19, 20), but hypertension may also be present in early stages of acromegaly, entailing a key negative prognostic factor of mortality (11).

Acromegaly-related hypertension is characterized by elevated diastolic blood pressure and a higher prevalence of non-dippers. Besides, it is usually not related to gender or family history of hypertension (18–23), although data from the recent Mexican registry has reported a higher prevalence in women (24). The relationship with IGF-I levels seems to be positive when these are frankly elevated, but not if acromegaly is relatively well-controlled (25). In fact, most studies have not found that GH or IGF-I levels at diagnosis correlated with the presence of hypertension (19, 26, 27).

The potential mechanisms involved in the development of acromegaly-related hypertension are still not fully elucidated, but include the expansion of plasma volume and sodium and water retention, stimulation of smooth muscle cell growth, increased vascular responsiveness to angiotensin II, increase in cardiac output, and increased peripheral resistances (20–25). Most of these effects are due to chronic GH-IGF-I excess, but a potential contribution of an increased sympathetic tone and the potential role of an increased vascular growth factor have also been suggested (20, 28). Furthermore, coexisting comorbidities, such as cardiac hypertrophy and/or sleep apnea, may exacerbate established and long-standing hypertension (20).

### Cardiomyopathy

Estimation of the prevalence of cardiomyopathy in general population is somehow difficult, but it has been reported to affect around 1 in 500 individuals (29). Myocardial hypertrophy may occur prematurely during the course of acromegaly and worsens with disease duration and coexistence of other classical cardio-vascular risk factors. However, a specific acromegaly-related cardiomyopathy has also been described in this particular setting; in fact, cardiac alteration may be found in patients with acromegaly who do not have any of the other cardio-vascular risk factors, and, thus acromegalic cardiomyopathy has been considered a hallmark feature of the disease (30). These results from direct effects of GH and IGF-I on the heart, rather than from processes associated with lipotoxicity, since active acromegaly seems to protect from ectopic lipid accumulation in the heart and liver (31).

The pathophysiologic mechanisms involved in the development of cardiac alterations include an increased deposition of collagen and lymphomononuclear cells, which lead to architectural modifications (hypertrophy, degeneration, and fibrosis) and alterations in water balance, and subsequent cardiac hypertrophy, leading to diastolic and systolic dysfunction, low cardiac output, and eventual heart failure (20, 30, 32–42). Specifically, we can differentiate a first stage, mainly characterized by a hyperkinetic left ventricle that causes increased contractility and cardiac output; a second stage of progressive hypertrophy, fibrosis and diastolic filling deterioration; and a third stage in which both diastolic and systolic dysfunction evolves to subsequent clinical congestive heart failure (28, 30).

Acromegalic patients with cardiomyopathy are usually diagnosed in the second phase, when their compromised diastolic filling and function affects his/her exercise capacity (30). Although the actual prevalence of cardiac failure is low in acromegaly, the global prognosis when patients develop chronic congestive heart failure is poor (43).

Age, hypertension, disease activity, and duration have been identified as potential risk factors for developing left ventricle hypertrophy in acromegalic patients (30, 38, 44, 45). However, some authors have questioned the true role of hypertension in the specific setting of acromegalic cardiomyopathy, since they did not find differences in the prevalence of left ventricle hypertrophy between patients with and without hypertension (37, 46).

### Heart Valve Disease

Mitral and/or aortic valves are the most frequently affected ones in the particular case of acromegaly due to chronic GH/IGF-I excess. Whilst heart valve disease has been reported to occur in general population a wide age-related range of 0.7–40% (47, 48), cardiac valvulopathy may affect up to 75% of acromegalic patients at the time of diagnosis (49). It is a result of an imbalance in the regulation of the extracellular matrix and the deposition of collagen and mucopolysaccharides at the level of valvular leaflets, which leads to leaflet disarray, valve ring fragility and valve regurgitation (50–52). Aortic root ectasia may also develop as left ventricular hypertrophy progresses (53, 54).

Once again, disease duration seems to be directly associated to the severity of valve disease, which includes ring fragility, leaflet disarray, and valve regurgitation (49–52, 55, 56), and may exacerbate concomitant heart abnormalities.

### Arrythmias

Several types of arrhythmias, including paroxysmal atrial fibrillation and supraventricular tachycardia, sick sinus syndrome, isolated and paired ventricular ectopic beats, and ventricular tachycardia, have all been linked with acromegaly (57, 58). However, 24 h Holter and cardiac magnetic resonance study have not proven that these arrhythmias entail clinical relevance (59).

In addition, left ventricular dyssynchronicity may also be present in acromegalic patients. This particular rhythm abnormality consists of the loss of the simultaneous peak contraction of corresponding cardiac segments. It is worth mentioning that this cardiac alteration occurs independently from typical predictors of cardiovascular disease in acromegaly, such as age at diagnosis, disease duration and coexistence of other cardiovascular complications. Therefore, a potential direct hormone effect on cardiac synchronicity, associated to disease activity is presumed (28, 59–61).

### Atherosclerosis and Coronary Artery Disease

The prevalence of atherosclerosis in acromegaly has been a matter of debate and controversy across several reports. For instance, in some studies, an increased carotid intima-media thickness has been reported in patients with active acromegaly, compared to matched healthy controls, or patients with inactive acromegaly (21, 62, 63). Another study, however, did not evidence an increased prevalence of coronary artery disease, carotid atherosclerosis, or increased carotid intima-media thickness in comparison to normal subjects (64). Further retrospective studies, including national registries, have reported rates of coronary heart disease ranging from 2.5% in an Italian cohort (5), 7% in Belgium (65), 8% in a tertiary center in Mexico (66), and 12% in France (67), denoting the wide variety, and heterogeneity in the prevalence of this complication in acromegalic patients. This is rather similar to what occurs in general population, in whom the prevalence of atherosclerosis and coronary artery disease has also been variable depending on the diagnostic method used for its definition, and the population studied, given the influence of age, sex, and the presence of other concomitant cardiovascular risk factors.

The risk of coronary artery disease (as evaluated by the Framingham Score) and the prevalence of coronary atherosclerosis (assessed by quantification of coronary artery calcium in cardiac computed tomography) have been reported as high, medium, or low (32, 52, 68). This fact suggests that acromegaly does not imply intrinsically an additional risk on top of known coronary artery disease risk factors (69–71). Intriguingly, in acromegaly, the concomitant presence of additional classical cardiovascular risk factors, such as smoking, sleep apnea, insulin resistance, hyperglycemia, dyslipidemia, and excess body weight, seem to have a greater influence on the development of coronary disease and atherosclerosis, rather than chronic and long-term exposure to GH/IGF-I excess (68). Alternative methods measuring endothelium dependent dilatation and microvascular function (72) may provide new data in the future.

In any case, even though it is not a particular and specific mechanism occurring exclusively in the setting of acromegaly, the main underlying mechanism for vascular damage in these patients involves endothelial dysfunction and atherogenesis, insulin resistance, vascular hypoxia due to obstructive sleep apnea, and oxidative stress. Insulin resistance impairs endothelial nitric oxide synthase and nitric oxide formation, and stimulates mitogenic insulin pathway, and thus, an increase in the synthesis of endothelin-1 (a marker of endothelial dysfunction) in endothelial cells, with a potential additional effect of GH excess (68, 73, 74).

## How Can We Diagnose Cardiovascular Comorbidities in Acromegalic Patients?

Despite numerous consensus conferences and expert meetings aiming to elaborate specific recommendations for the management of acromegalic patients, a clear endorsement for this particular setting has not yet been achieved. Thus, there is no definite consensus on the examinations that should be performed and the timing for performing them, and only general recommendations have been made. (75, 76). The most recent consensus statement on acromegaly therapeutic outcomes (15) discretionarily recommends that clinician-reported outcome instruments such as SAGIT (Signs and symptoms, Associated comorbidities, GH levels, IGF1 levels, and Tumor profile) (13) and ACRODAT (Acromegaly Disease Activity Tool) (14) could be potentially used to assess and monitor indicators of disease activity. However, these tools are confined to recognizing if comorbidities are present or not, but do not explicitly describe how these comorbidities should be diagnosed.

A summary of the diagnostic tests that may be used to identify the different cardiovascular alterations is depicted in Table 1. The method of choice and the frequency it should be done is controversial.

**Table 1:** Summary of the diagnostic tests that may be used to identify the different cardiovascular alterations in patients with acromegaly.

**Diagnostic test**	**Utility**
- Manual office blood pressure measurements - Repeated home blood pressure measurement - 24-h ambulatory blood pressure measurement (ABPM) - Automated office blood pressure (AOBP) measurement	Hypertension
- Electrocardiography (ECG) (single clinic 12-lead ECG) - 24-h Holter ECG monitoring	Arrhythmias Cardiomyopathy
- Exercise, tredmill - Stress tolerance test - Echocardiography - Ultrasound and duplex study of carotid and supraaortic trunk, including intima-media thickness measurement, quantification of internal carotid stenosis and number, morphology and surface characteristics of carotid plaques - Cardiac magnetic resonance imaging (cardiac MRI)	Arrhythmias Cardiomyopathy Coronary artery diseases Heart valve disease
- Coronary computed tomography angiography - Coronary catheterization - Positron-emitted tomography,	Cardiomyopathy Coronary artery diseases

It is worth remarking that, although echocardiography was the traditional standard technique to evaluate heart hypertrophy, more recent studies tend to use cardiac magnetic resonance imaging (MRI). So findings regarding cardiac morphological alteration in more recent studies may be determined by this shift in the diagnostic method used. In addition, in order to overcome the effects of variations in the patient's body weight, indexing the left ventricular mass for height powered to 2.7 seems to improve the accuracy when aiming to identify left ventricular hypertrophy in acromegalic individuals (30, 77).

Table 2 suggests possible screening methods for each of the potential cardiovascular comorbidities encountered, with its main *pros* and *cons* (8, 37, 41, 46, 58, 60, 76, 78–80).

**Table 2:** Possible screening methods for each of the potential cardiovascular comorbidities encountered in patients with acromegaly, describing their main pros and cons.

	**Advantage**	**Disadvantage**
Routine measurement of blood pressure during the programmed out-patient visit (“office measurement”).	Simple, non-invasive, short duration. May be collated with ambulatory blood pressure monitoring	May overestimate the prevalence of hypertension (16).
Electrocardiography (could be the one prescribe as part of the preoperative study) Assessment of QT intervals (77–80) Registration of late potentials. Further 24 h monitoring if abnormal initial punctual screening.	Identifies patients with higher risk of rhythm disorders (50, 52)	Prognostic value in the specific setting of acromegaly has not been fully evaluated
Echocardiogram (70)	Non-invasive. High resolution for ventricular anatomy and function. Useful to assess the severity and extent of acromegalic cardiomyopathy (8, 68). Moderate cost. Good reproducibility.	Calculation of the left ventricle mass uses a cubing formula, so small errors may be amplified and left ventricle mass may be over or underestimated (77).
Echocardiogram with pulse issue Doppler	At early stages may identify subclinical biventricular impairment of systolic function (75).	Same issues as for echocardiogram.
Radionuclide angiography	Non-invasive assessment of rapid diastolic filling. Evaluates the integrity of patients' cardiac performance.	Requires injection of radionuclide into vein. Cost.
Gadolinium-enhanced magnetic resonance imaging (MRI)	Gold standard. Higher accuracy and reproducibility and lower variability than echocardiography (71, 72). Myocardial transverse relaxation time (T2) allows a non-invasive assessment for detecting myocardial edema, and thus the direct action of GH and IGF-1 on the heart. Serves to evaluate the efficacy of acromegaly treatment regarding cardiomyopathy (35).	Less available. Cost. Inconsistent cost-effectiveness (31, 40)

Regarding atherosclerosis and coronary artery disease in the particular setting of acromegaly, because of the heterogeneity in the prevalence reported across studies, establishing the most optimal screening tools seems more complex (81–88). In this regard, if the occurrence of this particular cardiovascular comorbidity in acromegaly does not differ significantly from the one in the general population, there does not seem to be the need for a special program for diagnosis and surveillance of coronary artery disease in acromegalic patients. Thus, screening tools and timings should be performed based on the individual's cardiovascular risk profile (Framingham score) (64, 70, 71, 89). Specifically, for instance, a simple electrocardiogram, or a stress and/or exercise tolerance test, could help in the assessment of cardiac performance, when clinically relevant atherosclerosis is suspected. Nevertheless, there are also other alternative methods that allow the evaluation of arterial damage and could provide further information when deemed necessary in certain circumstances; for instance, applanation tonometry, pulse wave velocity, augmentation index, epicardial fat thickness, carotid ultrasonography intima-media thickness, aortic stiffness, flow-mediated dilatation, computed tomography angiography, coronary catheterization, positron-emitted tomography, or serum cell adhesion molecules (21, 28, 72, 89, 90).

A recent update on the diagnosis and treatment of acromegaly complications (76) recommended to perform an electrocardiogram, echocardiogram, blood pressure measurement, and the Epworth scale or sleep study for sleep apnea, at baseline, and then every year, in patients with newly diagnosed acromegaly. This could be even considered as part of the preoperative study an acromegalic patient may face prior to undergoing transesphenoidal surgery. In addition, assessment of the peripheral arterial system may be performed to fulfill a thorough evaluation and rule out the presence of vascular disease.

In our country, a recent national consensus of experts (91) endorsed that screening for hypertension and cardiovascular disease should be performed routinely at the time of diagnosing acromegaly. The former should be screening using the usual “office measurement,” and ABPM or AOBP if doubtful. The latter should be ruled out with a simple routine electrocardiogram, but, again, 24 h Holter should be performed if there is the suspicion or graphical evidence of rhythm disorders. No clear consensus was reached regarding the need for routine echocardiogram at the time of initial diagnosis of acromegaly, nor if it was truly necessary in young acromegalic patients. However, an initial echocardiogram was indeed recommended in cases of acromegalic patients with concomitant hypertension and/or diabetes, with subsequent follow-up accordingly. Besides, a stress tolerance test should probably only be performed if ischemic heart diseases is suspected.

In any case, what is truly important is to maintain longitudinal monitoring and rigorous management of individual complications during long-term follow-up (8). For instance, in the aforementioned national consensus (91), experts agreed that hypertension and cardiovascular disease should be ruled out every 6–12 months in cases of active acromegaly, or when directed treatment was modified.

## How Does Disease Control Affect Cardiovascular Comorbidities In Patients With Acromegaly?

**Figure 2:**
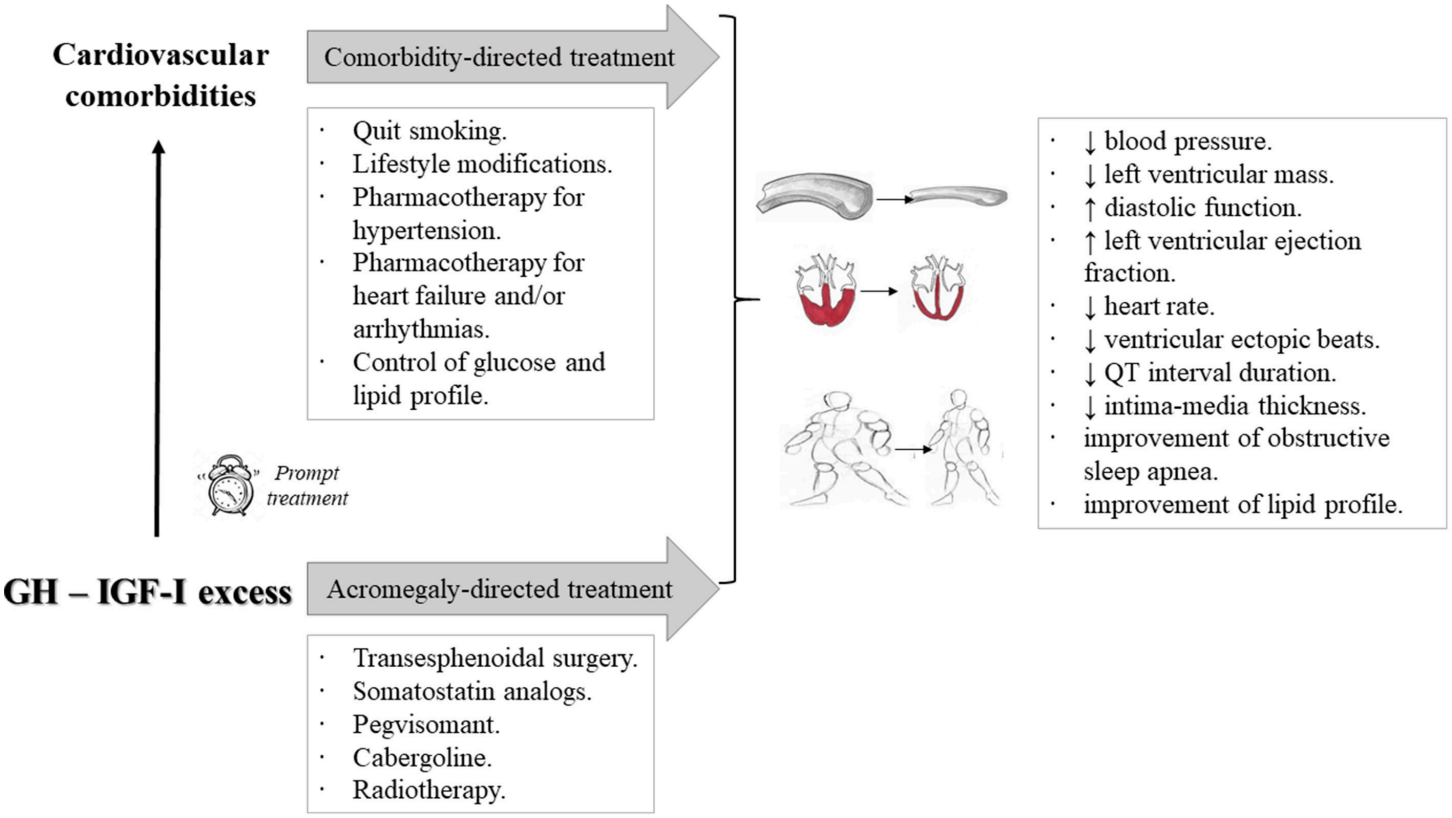
Schematic representation of the therapeutic approach to acromegalic patients with cardiovascular comorbidities and its reported effects. It is worth emphasizing that a prompt initiation of treatment allows a higher probabilitiy of cardiovascular control. In fact, regression of established alterations may be even feasible. A general management should be warranted, including smoking cessation, lifestyle modifications, and optimal pharmacotherapy for each of the potential problems encountered, including adequate control of glucose and lipid profiles. Besides, control of GH/IGF-I excess with the available acromegaly-directed treatment modalities further ameliorates cardiovascular comorbidities. ↓: decrease; ↑: increase.

Given the fact that GH/IGF-I excess plays a role in the development of cardiovascular comorbidities in patients with acromegaly, it seems reasonable to hypothesize that disease control may ameliorate them (Figure 2). In fact, two large comprehensive reviews exploring this issue concluded that control of GH and IGF-I excess could halt the progression of cardiovascular abnormalities, as well as the observed morphological and functional cardiac alterations (58, 89). Additionally, concomitant cardiovascular risk factors, such as hypertension, dyslipidemia, diabetes mellitus, and sleep apnea syndrome, could also improve (28, 92–94). Epidemiological data have confirmed these findings, and disease control in acromegalic patients has been found to be one of the most important determinants of patients' outcome (5), and consistently associated to a reduction in cardiovascular mortality (1, 2, 55). In fact, this conclusion has also been reached in another large meta-analysis (7).

To answer the question if all comorbidities respond equally to disease control, a few considerations must be pointed out. It is true that a strict biochemical control of acromegaly deems necessary for an optimal treatment, or even prevention, of concomitant cardiovascular complications. However, age at diagnosis, smoking, and overweight/obesity are further influencing factors, as in the rest of the general population, and should not be overlooked (5). In this regard, the relationship between control of acromegaly and reversibility of cardiovascular comorbidities may seem paradoxical; for instance, some cardiac alterations are not reversible despite an adequate control of GH/IGF-I levels, whilst other comorbidities may improve significantly with treatment of acromegaly, even if GH/IGF-I levels remain elevated. The latter would be the case, for example, of cardiomyopathy: indeed, although a greater improvement was demonstrated in patients with normalized IGF-I, benefits were also evidenced in patients with “only” biochemical improvement. This could be relevant for clinicians' biochemical targets to decrease cardiovascular complications, which may not necessarily be the same as those for biochemical normalization (95–97).

On the other hand, when interpreting the effects of treatment of acromegaly on cardiovascular parameters, we must be aware of the fact that patients' characteristics and the diagnostic methods used across studies may be heterogeneous, entailing certain differences, subtle or not, in the prevalence of comorbidities and, thus in the drawing of conclusions. For instance, some studies used echocardiogram, but others deployed cardiac MRI, so the evaluated effects on left ventricular hypertrophy may be discordant (37, 46, 95).

In any case, ongoing or development of new cardiovascular complications should still be monitored in patients who do fulfill achievement of optimal biochemical treatment targets (35).

### Approach to Cardiovascular Comorbidities in Acromegaly: General Aspects

Management of hypertension is similar to routine management. In fact, standard treatment with anti-hypertensive agents, such as thiazide-type diuretics, angiotensin converting enzyme inhibitors (ACEI), angiotensin II receptor blockers (ARB), calcium channel blockers, or even amiloride, is usually recommended (1, 8, 76, 91). In addition, a prompt and adequate management of sleep apnea syndrome may help in achieving optimal blood pressure control (77, 92). Acromegalic cardiomyopathy can be reversed if diagnosis was early in the course of the disease (1, 38, 39, 41, 98, 99). Established valve damage, however, seems to be irreversible, although further worsening may be prevented if acromegaly is adequately controlled (11). Atherosclerosis, on its side, may be better managed by targeting commonly recognized cardiovascular risk factors (for instance, smoking status, insulin resistance, hypertension, etc.) (8).

### Acromegaly-Directed Treatments and Their Effect on the Outcome of Cardiovascular Comorbidities

Effective transesphenoidal surgery of the pituitary adenoma reduces left ventricular mass and improves cardiac function (100, 101).

Somatostatin analogs (SSA) may also improve cardiovascular parameters in a significant way (89). For instance, they reduce blood pressure (101, 102), improve heart rate and cardiac function, reduce left ventricular mass, improve systolic and diastolic function and exercise tolerance (101–104), improve diastolic filling (96), and may also reduce the occurrence of arrhythmias (105, 106), although their effect on heart valve disease is less relevant (49, 107, 108). In general, the effects of SSA seem more significant when used for periods longer than 6 months, especially in patients in whom control of GH/IGF-I excess is attained (101, 109). In this regard, if efficacy of SSA on cardiovascular comorbidities depends on their benefit in controlling acromegaly itself, a prompt diagnosis will allow early initiation of treatment and a potential arrest, or even regression, of morphological and functional cardiac abnormalities (82). Some studies have reported the expression of somatostatin receptors type 1, 2, 4, and 5 in atrial and ventricular tissue, suggesting the possibility of a direct effect of SSA on the heart (104, 110). In fact, in a relatively large open-label, randomized study of patients with recently diagnosed acromegaly, treatment with SSA had beneficial effects on cardiomyopathy that were not observed in patients who underwent surgery. Interestingly, in this series, the rate of successful control was similar (111). Likewise, systolic function, expressed as ejection fraction, improved more evidently in SSA-treated patients exclusively in a retrospective comparative non-randomized study (112). The deterioration of glucose homeostasis potentially exerted by SSA, particularly for the newer pasireotide, must be acknowledged, in an aim to keep comorbidities as strictly controlled as possible despite this potential adverse effect (113–118). It is worth remarking that the utility of primary treatment with SSA has been addressed in several consensus guidelines as a potential preoperative tool in selected patients with evident cardiovascular alterations, including cardiomyopathy, heart failure or arrhythmias, because, in such patients, improvement of cardiac function may enhance anesthetic safety (8, 91, 105).

Pegvisomant, on its side, may likewise ameliorate cardiovascular comorbidities, following its efficacy in controlling IGF-I excess (119–123). In fact, treatment of acromegalic patients with pegvisomant improved left ventricular mass, systolic and diastolic function and blood pressure, reduced the prevalence of conduction disturbances, and even decreased the Framingham risk score after 12 months of treatment in the German Pegvisomant Observational Study (121, 123–125). Furthermore, the reduction in levels of glucose, insulin, and HOMA index may be relevant for its overall beneficial role (94, 119, 120), because of the collateral effect on glucose homeostasis.

Combination treatment with SSA and pegvisomant has also proved to be beneficial regarding cardiovascular outcomes. In fact, cardiac structure and performance, cardiac hypertrophy, and diastolic dysfunction, measured using different methods (ejection fraction, early to late ventricular filling velocities and isovolumetric relaxation times, for instance), significantly improved after long-term combined therapy (97).

Regarding the dopamine agonist cabergoline, to our knowledge, there are no specific studies evaluating its role in the outcome of cardiovascular comorbidities in patients with acromegaly. Even though there are several concerns regarding the potential development of heart valve alterations with the use of dopamine agonists, the relevance in acromegalic patients is still unclear because the doses used in these cases are usually lower (126). Nonetheless, although valve complications are not frequent in patients receiving conventional doses for pituitary tumors, it is essential to monitor periodically with serial echocardiography those patients who receive higher than conventional cabergoline doses for prolonged periods (127–129). Moreover, as a precautionary measure, cabergoline should probably not be used if pre-established valve disease is present (91).

For radiotherapy in patients with acromegaly, to our knowledge, there are still not enough studies that focus on the influence of this treatment modality on cardiovascular comorbidities. This may be because radiotherapy has usually a delayed effect, and the concomitant risk of pituitary deficiency does not facilitate a reliable analysis of its beneficial effects on cardiovascular risk (130, 131). Besides, pituitary radiotherapy may be associated to the development of cerebrovascular disease (20, 132, 133).

## Conclusions

Cardiovascular comorbidities are frequent and paramount in acromegalic patients. Their importance relies on the meaningfully increased risk of morbidity and mortality that they entail. Thus, a prompt and thorough evaluation deems necessary to adequately manage them, in addition to management of GH excess itself. The underlying physiopathologic mechanisms for each comorbidity may not be completely understood; in fact, there seem to be mechanisms similar to those that also occur in general population, but, in addition, persistent GH/IGF-I excess, age, prolonged disease duration, and coexistence of other cardio-vascular risk factors, behave as key predisposing factors. It is essential to rule out the presence of hypertension, cardiomyopathy, heart valve disease, arrhythmias, atherosclerosis, and coronary heart disease, and therapeutic strategies should be approached accordingly. Besides, other classical cardiovascular risk factors, such as smoking, sleep apnea, insulin resistance, hyperglycemia, lipid disorders, and obesity need to be continuously addressed and appropriately targeted. An initial diagnostic approach could include baseline assessment of blood pressure and an electrocardiogram, and maybe an echocardiogram in certain particular cases. Then, depending on findings from initial tests, further diagnostic procedures could be required on an individual basis. Rigorous clinical follow-up, long-term monitoring, adequate GH/IGF-I control, and continued emphasis on lifestyle modifications remain important mainstays of management strategies.

## Author Contributions

AR-L and MM researched data, wrote the manuscript and approved its final version.

### Conflict of Interest Statement

AR-L has received lecture fees from Ipsen and Novartis. MM has received lecture and advisor fees from Pfizer, Novartis, and Ipsen.
